# Women Jurors

**Published:** 1921-01-29

**Authors:** 


### Women Jurors.

An extraordinary amount of public interest has been aroused in the first appearance of women in the jury box in courts of law in this country. Why it should be so is not easy to determine. A good deal of nonsense has been said and written about the fundamental difference of outlook in public affairs between the two sexes, and we might almost suppose from some of the printed comments that women jurors may be expected to revolutionise the whole spirit of British judicial procedure. There is nothing in human nature or sex psychology which justifies these expectations. The woman juror is in logical succession to the woman voter. Serving on juries is part of her new responsibilities in public affairs; it is an ordinary right and duty of citizenship, just as serving on town councils, on health committees, in public departments of State, and in all the other social activities of the nation is a right tardily recognised after centuries of sex-prejudice. Women themselves for the most part would probably resent the suggestion that they will be a failure if they do not within a reasonable time introduce a new heaven and a new earth into the terrestrial scheme of things. What they will do is to divide the burden of responsibility with men, and share the problems of government on an equal footing. That in itself is of considerable importance, because it will restore the balance of sex, and contribute to a harmonious and more rational administration. Already the women jurors have shown that their standard of intelligence is not essentially different from that of their male colleagues; and neither emotion nor misdirected sentiment seems to have played any part in deflecting their judgment in the discharge of their new duties. Indeed, jurymen have in one or two cases acknowledged the assistance which they have received from women members in coming to a sound decision in accordance with the evidence. It is too readily assumed that woman's modern contribution to the cause of humanity and progress will be solely the emotional factor. One prefers to hope, in view of the help they have already given, that it will be an addition to the sum of our common-sense.

